# Attitudes among Parents towards Return of Disease-Related Polygenic Risk Scores (PRS) for Their Children

**DOI:** 10.3390/jpm12121945

**Published:** 2022-11-23

**Authors:** Shannon Terek, Maya C. Del Rosario, Heather S. Hain, John J. Connolly, Meckenzie A. Behr, Margaret Harr, Hakon Hakonarson, Ingrid A. Holm

**Affiliations:** 1 Center for Applied Genomics, Children’s Hospital of Philadelphia, Philadelphia, PA 19104, USA; 2 Division of Genetics & Genomics, Department of Pediatrics, Boston Children’s Hospital, Boston, MA 02115, USA; 3 Children’s National Hospital, Rare Disease Institute, Washington, DC 20010, USA; 4 Department of Pediatrics, The Perelman School of Medicine, University of Pennsylvania, Philadelphia, PA 19104, USA; 5 Division of Human Genetics, Children’s Hospital of Philadelphia, Philadelphia, PA 19104, USA; 6 Division of Pulmonary Medicine, Children’s Hospital of Philadelphia, Philadelphia, PA 19104, USA; 7 Harvard Medical School, Boston, MA 02115, USA

**Keywords:** pediatric, polygenic risk scores, return of results, diverse population, semi-structured interview

## Abstract

The electronic MEdical Records and GEnomics (eMERGE) consortium will return risk reports pertaining to specific diseases, a key component of which will be polygenic risk scores (PRS), to 25,000 participants, including 5000 children. Understanding comprehension and the perceived value of these PRS-based reports among parents will be critical for effective return of results in children. To address this issue, we conducted semi-structured interviews with 40 African American and Hispanic parents at The Children’s Hospital of Philadelphia and Boston Children’s Hospital. Each participant received a hypothetical risk report identifying their child as high risk for either type 2 diabetes or asthma. Participants were assessed on their comprehension of absolute versus relative risk framing, likelihood of following risk-reduction recommendations, perceived value of the information, psychosocial impact, education/support needed, and suggestions to improve the PRS-based report to make it more accessible. Results demonstrated high perceived value in receiving PRS-based reports but also draws attention to important shortfalls in comprehension due to factors including the health of the child, family history, and how the risk was framed. This study provides an insight into implementing the return of genomic risk scores in a pediatric setting.

## 1. Introduction

The last several years have seen a surge of interest in the use of polygenic risk scores (PRS) to assess an individual’s disease susceptibility. PRS, which use genotype data to assess risk, have the potential to outperform clinical predictors for several diseases, including but not limited to breast cancer, type 1 diabetes, asthma, and prostate cancer [1,2,3,4].

Several studies have shown that simply providing people with their genetic risk information does not lead to improved health behavior [5,6]. Little is known about how individuals perceive risk, which may impact on whether they adopt medical and/or lifestyle changes to reduce risk. Early work by Kahneman and Tversky [7,8,9] highlights a range of cognitive processes that impact risk perception and can have major consequences on healthcare decisions [10,11]. One of the most important determinants on how risk is perceived is framing, whereby the format in which alternatives are presented can dramatically affect choices. Several framing paradigms have been shown to have major effects on risk determination and subsequent actions, yielding huge disparities between equivalent choices [8,12,13,14].

Barriers to acting on PRS information may also affect adoption of improved health behaviors and may include income inequality and health disparities. The ethical, legal, and social implications of reporting PRS, particularly when individuals do not have the means to change lifestyle, are important to consider [15]. Additionally, there may be misunderstanding of, and/or uncertainty about PRS [16]. Little is known about potential barriers, especially in children and families, and in minority populations that are under-represented in research.

To further understand how PRS could be integrated into clinical care, the electronic MEdical Records and GEnomics Network Phase 4 (eMERGE 4) was funded by the NHGRI/NIH to conduct a large-scale study to develop and validate PRS for common adult and pediatric diseases and study the medical outcomes and impact on providers and participants. The Network will target enrollment of racially and ethnically diverse and underserved populations, who have yielded comparatively less benefit from genomics research to date [15]. A major goal of eMERGE 4 is to study how individuals use PRS results to decrease their risk for disease through medical and/or lifestyle interventions, such as changes in diet and exercise. With this goal, the eMERGE 4 study prioritizes addressing the ethical, legal, and social implications (ELSI), including the development of a comprehensive suite of resources to support participants in understanding their genomic information and potential impact on clinical care.

To inform the development of return of results materials and processes in the pediatric setting, we examined the perceived value of returning genomic risk estimates in children to their parents and investigated factors influencing that perception. We conducted semi-structured interviews with parents who received hypothetical risk reports on their child to assess their (1) understanding of PRS-based hypothetical reports, (2) overall risk perception, (3) barriers to following report recommendations, and (4) education/support needed. We aimed to explore whether factors such as risk framing, overall comprehension of the PRS and report, and the ease of implementation of risk-reducing recommendations influenced parental perceptions of genetic risk estimates. The goal was to obtain data to develop best practice recommendations for the return of genomic risk estimates in children. Ultimately, these findings will be used to inform the development of reports returning PRS-based risk to parents.

## 2. Materials and Methods

### 2.1. Participants

Participants were parents/legal guardians of children enrolled in the Center for Applied Genomics at The Children’s Hospital of Philadelphia (CHOP) or the Precision Link Biobank at Boston Children’s Hospital (BCH). Patients at each institution were offered voluntary enrollment to the biobanks for research study purposes from trained study staff who approached patients during hospital stays or routine-care visits in accordance with site-specific IRB protocols. Those who were enrolled in the biobank had the option to indicate that interest in hearing about future research studies, if eligible, by being re-contacted. Those who had enrolled in the biobank within the previous two years, and who had given permission for re-contact, were contacted by the recruitment team(s) and given information about this study. Because of the eMERGE 4 Network’s focus on enrollment of traditionally underrepresented individuals, parents of Black/African American or Hispanic ancestry were contacted. To avoid bias in the interviews, those individuals with known diagnoses of type 2 diabetes and/or asthma were excluded. We were otherwise naïve to the medical history of participants’ children. The study was approved by the Institutional Review Boards (IRBs) at CHOP and BCH.

### 2.2. Hypothetical Risk Report

**Figure 1:**
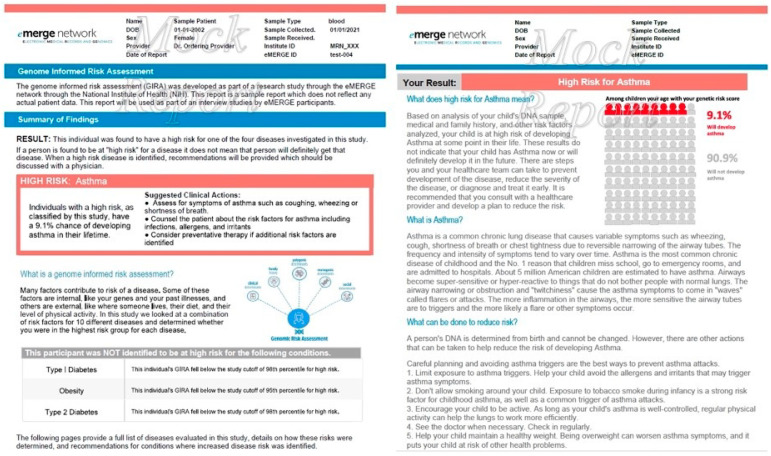
Mock GIRA report for high risk asthma presented as an absolute risk. The report is modeled off the return of results report to be used in eMERGE 4.

To control for order effects, participants were randomized to receive a hypothetical risk report called “GIRA” or “Genome Informed Risk Assessment” based on the GIRA planned to be returned in the eMERGE 4 study (Figure 1). The reports included a summary of findings (e.g., high risk estimates for asthma or type 2 diabetes (T2D)), explanation of what the genome informed risk assessment is and means, what they were not found to be high risk for, explanation of the condition, and what could be done to decrease the risk (primarily lifestyle changes).

**Figure 2:**
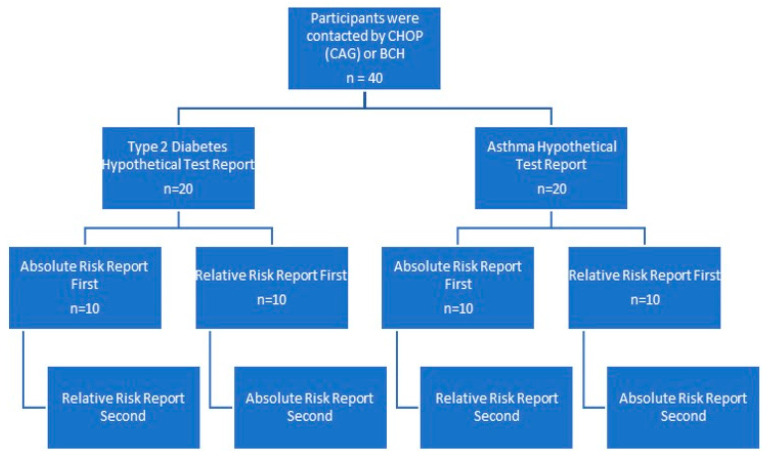
Workflow of conducted interviews.

Within each condition, participants additionally were randomized to receive the hypothetical risk estimates presented as either (1) absolute risk (e.g., “Your child has a X% risk of developing T2D/asthma within 10 years”.) or (2) relative risk (e.g., “Your child has a X times increased risk of developing T2D/asthma within 10 years”.). Half of the participants were shown the absolute risk reports first, and half were shown the relative risk report first. They were then presented with the alternate report (absolute or relative), allowing them to compare the two methods of displaying risk (Figure 2).

### 2.3. Interviews

Two interview guides were developed, one for a hypothetical risk report for T2D and one for asthma (see Figures S1 and S2) During the interviews, all participants were probed on their understanding of, and reaction to, the report. A response was considered misunderstood if the participant’s expression of risk was misaligned with the risk in the report document (i.e., was higher or lower). In order to elicit emotional responses, the interviewer asked, “How did this hypothetical report on your child make you feel?” If participants did not offer an emotional response, the interviewer asked the following probe: “For example, did you feel anxious, frustrated, curious, surprised, worried, overwhelmed, confused, confident, neutral?” Interviews were conducted by videoconferencing with Zoom or WebX, audio-recorded, and participants were compensated with a USD 50 gift card for their time. Audio recordings were transcribed, deidentified, and uploaded to the online qualitative analysis tool Dedoose (https://www.dedoose.com, accessed on 22 July 2022). Codes were used to identify participants in this study and then shortened for readability. The first letter indicates what site the participant was from (B is BCH, C is CHOP). The second letter indicates what condition the participant was high risk for (A is asthma, D is type 2 diabetes). The third letter indicates which risk framing method the participant saw first (A is absolute, R is relative). The following numbers are unique study identification codes to differentiate participants during coding.

### 2.4. Coding

Qualitative coding was used to systematically categorize themes and patterns based on content analysis of the interviews. The analysis team (i.e., study authors) used a deductive coding approach, which leverages codes developed a priori by the team (versus inductive coding that identifies themes through ongoing bottom-up analysis). The codebook was refined via preliminary coding of the first four interviews by two study team members (M.C.D. and S.T.). Discrepancies between the coded interviews were reviewed and adjustments to the codebook were made throughout the process. Once the final codebook was developed, coding was completed separately by the two coders (M.C.D. and S.T.). Each coder coded half of the remaining interviews, from both sites. After initial coding was completed, each coder reviewed the other coder’s interviews for reliability. Coders then worked together to review and identify discrepancies between datasets, which were ultimately merged.

## 3. Results

### 3.1. Study Population

Each site conducted 20 interviews for a total of 40 interviews (Figure 1). There were 10 participants (5 from each site) in each group: (1) T2D, absolute risk first, (2) T2D, relative risk first, (3) asthma, absolute risk first, and (4) asthma, relative risk first. Participants primarily self-identified as African American (87.5%), were female (97.5%), and were 31–40 years of age (50%). Most had at least some college education (77.5%). Over half had insurance through an employer or union, and 32.5% had some type of government-sponsored insurance. A little over a third (37.5%) had previous experience with genetic testing (Table 1). All participants were parents or legal guardians of children under the age of 18.

With a small sample size (*n* = 40), and relatively small effect sizes, we were not sufficiently powered to attempt quantitative analyses of the six Likert-based questions listed in the codebook. As such, the results below are based on qualitative analyses of the study interviews. Table 2 outlines the domains and themes discovered in the interviews and discussed below (Table 2).

### 3.2. Understanding of Report

The indication of the child’s high risk and increased susceptibility to each condition was largely, but not universally, understood by participants. Various factors including risk framing, family histories, and medical complexities of their child influenced how the risk scores were perceived.

#### 3.2.1. Absolute Risk Framing Was Preferred

When given risk displayed in two different formats, 50% of participants preferred absolute risk, while 37% of participants indicated a preference for relative risk (see Table 3) (see Table S1 for a further breakdown on risk preferences) Participants generally expressed that absolute risk was more familiar, relatable, and used in daily life, “*I like percentages, because I think that’s something we all better understand. We use more daily the percentage aspect*” (BAA09). Additionally, knowing exactly where their child stands in their risk to develop the condition was something that was very important to the participant, and this was something perceived as more easily accessible using absolute risk, “*It’s more helpful with the number of the actual percentage, because as a parent, I feel that knowing the exact amount would probably be better to obtain the information*” (BAA06).

#### 3.2.2. Absolute Risk Tended to Be Perceived as Low Risk

Although most participants preferred absolute risk framing, the percentages were often perceived as indicative of low risk. For T2D and asthma, respectively, the framing of “2% risk” and “9.1% risk”, which the eMERGE Network defined as “high risk,” often was perceived differently by participants, “*It’s concerning, but I feel like this is a lower risk…which then would make me question, why they called it a high risk?*” (BDA04). Thus, there was often a lack of concordance in risk perception between participants in this study and the eMERGE Network. Other participants interpreted the high risk result as low/average. One participant commented on the risk inherent in the (reported high) T2D result by saying, “*I would say, average. Because sometimes just because he has genetic predisposition doesn’t mean that your child will actually get the disease*” (BDA19). Due to the common nature of these conditions, there was some confusion as to whether the absolute risk was even higher compared to the general population, “*I guess if I were reading this I would say, wouldn’t everybody be 2%?*” (CDA23). It is important to note that baseline risks were not included on the hypothetical reports, causing participants to rely on various other factors and experiences that play into risk perception.

#### 3.2.3. Factors Contributing to the Risk Perception

Family History: Participants with a strong family history of the condition expressed that the high risk report was warranted based on their family history, “*I don’t have it, but my sister and aunts have it, and my husband has it, so I figure she has it on both sides. It would be high*” (CAA30). Having close family members with the condition lead participants to state that their children were automatically at a higher risk.

Trust in primary care providers (PCP)/pediatrician and hospital: Seeing that the report was from a health care system and/or doctor that they trusted led some participants to accept the report at face value. One participant stated, “*I trust her judgement, which is great. So as long as I trust her, I don’t have to worry about what she has to tell me*” (CDA22).

Children with multiple medical conditions: Although we did not ask participants about their child’s medical conditions, some participants mentioned that they have a child with extensive health concerns and some stated that those existing health concerns supersede concerns about the risk report, “*My background of having a kid with medical conditions—we have very pressing medical conditions, and then we have medical conditions that I would go crazy if I had to constantly worry about. If I got this result about asthma, I’d be like ecch, I’m not going to worry about that*” (BAA05).

Role of environment: Many participants noted the multifactorial nature of the conditions (asthma and T2D) and that the risk for developing the condition was not dependent only on genetic risk, “*I mean it’s a lot of variables that they can or cannot get it, so it’s not definitive…it all depends on their genetics, and their surroundings, and just being around certain things*” (CAR32).

### 3.3. Risk-Reduction Steps

All participants reported willingness to take at least one risk-reduction step, which was consistent across phenotypes and reports. However, respondents highlighted barriers and needed resources.

#### 3.3.1. Behavioral Barriers to Taking Risk-Reduction Steps

Some participants expected a lack of motivation/cooperation in their child, including a reluctance to change diet or exercise habits (T2D), monitor blood sugar (T2D), or participate in certain activities (asthma/T2D). One participant said, “*I know it’s hard to get my child to eat more and trying to get him to—physically try to get them to eat more or to participate in certain activities”.* (CAR27). Several participants noted that having a child with a behavior disorder, such as ADHD or autism, could magnify these barriers, “*She’s autistic, so the behavioral issues of her, and the lack of motivation and cooperation with her*” (CDA29). Participants also noted that pressure from their child’s peers and stigmatization could diminish compliance with recommendations (e.g., use of inhaler or medications), “… *having a child feel different from their peers and stuff… I know that that can be a toll on kids, too. You know, like if they have to take an inhaler and stuff and the age group*”. (BAR06).

#### 3.3.2. Lack of Resources Was a Barrier

When discussing risk-reduction steps, participants highlighted difficulties in having the proper resources when it came to (1) affordable healthy food, (2) educational materials, (3) insurance, (4) in-home behavior services, and (5) physical activity/recreation. Barriers related to affordability/cost were expressed, where even relatively minor costs can be prohibitive. One participant stated, “*I hate my copays. I hate the $30.00 copay…that’s a barrier for many people. I know people who don’t go to the doctor because they don’t have the copay*”. (CDA23). Another participant summarized a phenomenon thought to affect many families saying, “*It’s sad that healthy food is more expensive than everything we shouldn’t eat. So, when you don’t qualify, like so many others, for food stamps, it digs a very, very deep hole in the pocket*” (CDR36). Lack of time was identified as a barrier, for example, to attend clinic, exercise, and cook healthy meals: “*Time is a resource, and you know, a resource that many of us do not have, especially if you have a child where you’re not able to get off. Your job doesn’t offer, you know, like medical leave or anything*” (CAA25). Not having a detailed plan with the PCP was also identified, which speaks to a desire for shared decision-making and more education with someone they trust, “*It would be good if, you know, the doctor would put a plan together, like Monday through Sunday*” (CDR34). Additional barriers/lack of resources include a perceived lack of control over what occurs in the care of others (e.g., another home), having other children (thereby straining resources/time), and problems applying too many changes at once (which may call for a certain pragmatism in promoting recommendations).

### 3.4. Reactions to the Report

#### 3.4.1. Negative Emotions in Response to the Report

The three most reported negative responses expressed by participants were anxiety (40%), worry (33%), and confusion (23%). [As discussed, participants were asked how the report made them feel, and in the few interviews where no emotional response was offered, they were given a list of potential responses as a probe.] Past experiences with the condition or having been previously told their child was high risk made participants concerned with the results on the report, “*When I look at diabetes, people who I know who have had it…that’s just because of my experiences. So, I would say a little anxious*”. (CDA23). Additionally, having no experience with this type of result and report made individuals feel overwhelmed by the amount of information and the impact associated with it, “*I can see feeling a bit of like overwhelmed and confused at first. Especially if you don’t take the time to stop and read through…I would definitely be anxious or worried*”. (CAA25).

#### 3.4.2. Positive Emotions in Reaction to the Report

The top three reported positive responses were curiosity (38%), relieved (28%), and confident (25%). Many participants felt this report was a way to reduce the chances of their child developing the condition and provided them with information to act in their child’s best interest, saying, “*It gives you a sense of relief, as well. I think that you have a report that is going to help you alleviate more risk*”. (BAR14). Specifically, the report section on “ways to reduce risk” made participants confident they had the proper resources to lower the risk and receive care, “*I like the fact that I’m confident in the things I can do to change…I’m confident I can fulfill it*”. (CAA35).

Although most participants had some sort of response to the report, many felt negative feelings at first, but after processing, felt more positively overall*, “When I first looked at it, the first thing that jumped out was the red section…which freaks me out. But then when you read more into it, it is not as scary as you think it is*” (BDR20). Seeing the words “high risk” and the colors associated with the report, made participants feel there was a high level of concern for their child. However, once participants were able to read through the report, several stated it made them feel at ease and empowered to help their child, “*It gave me hope because that way…it doesn’t have to be a sad situation where the illness defines her…it makes me think, okay, well now that I read this report, I can help my child make sure she’s healthy*” (CDA23).

### 3.5. Value of the Report

#### 3.5.1. Report Prepared Them to Take Action to Reduce Risk

While anxiety, worry, and confusion were common reactions, many participants expressed that seeing these kinds of reports would help them feel prepared to take actions to reduce their child’s risk for getting a disease, “*If once it does come up, you start doing research on it and you can prepare yourself for what’s ahead*”. (BAR06). Additionally, they considered genetic risk testing and receiving risk reports to be beneficial for understanding not only the potential risk for immediate family members, but also their own risk in the context of family history, “*If I got this report for my son I would then be concerned or already preparing for my daughter to possibly have the same genetic disposition*” (BDR04).

#### 3.5.2. Value of Primary Care Provider Explaining the Results

Recognizing participants may not understand everything on the report themselves, the majority (88%) stated that having their PCP explain the results of their genetic risk testing was highly valuable. Participants emphasized the value in having someone who is intimately acquainted with their child’s health, such as their child’s PCP, review the results of risk testing, “*A lot of time when you have questions and you need information… you count on them to be able to refer you to a specialist… to give you the information you need so that you can understand*”. (CAR27). Additionally, most participants stated that their child’s PCP would be able to clearly explain the results of the testing, monitor for signs of the disease, and they would trust their PCP’s recommendations to reduce risk. As one participant noted, having their child’s PCP review the results helped them feel confident moving forward with care, “*It’s very important because I don’t know what to look for, so with the primary doctor… they see different signs of multiple different things where they know to have it looked at, stuff that I may look at as something small and not important but to them, it’s something that may mean a whole lot in her development*”. (CDR36).

## 4. Discussion

As eMERGE 4 prepares to provide 25,000 individuals with genomic risk scores, and in particular 5000 children, it is imperative that we begin to develop best practice recommendations for the return of genomic risk estimates. In the context of return of results to children, making sure we understand parent perspectives, especially those from populations traditionally underrepresented in biomedical research, will contribute to a more well-rounded and equitable development of risk return practices for children.

The most common difference in understanding related to the interpretation of “high risk”. The eMERGE Network reported the results as “high risk” as the increased risk associated with the results is deemed by the Network to be clinically actionable, recommending lifestyle, medication, or surveillance changes. On the other hand, just under half of all participants did not interpret the risk of the condition, based on the result, to be “high” (30% saw the high risk as a low; 17.5% saw the high risk as average). Although the report with absolute risk framing was (moderately) more preferred by participants, reflecting a similar pattern in the literature [10,13], for many patients, the absolute risk was perceived as low or normal risk; some participants interpreted the absolute risk, although reported to be high risk, as average risk for the general population, likely because asthma and T2D are both common conditions. This puts into question the impact these reports will have on patient behavior. Other factors such as family history, trust in health care, medical history, and preferences in risk perception, seem to be important factors affecting how the report was understood.

Although the level of understanding of the report varied amongst participants, the expressed willingness to follow through on risk-reduction steps was consistent. When presented with ways to reduce the risk in their child, many parents stated they were certain they would follow through. This is similar to what has been seen in the past with the intention of changing behavior noted in many studies where risk information is shared with participants [6,17]. However, as previous studies have shown, intention does not represent whether behavioral changes occur, even when the genetic risk is high [6,17,18,19]. Many participants have not adhered to follow-up and care instructions partially due to the reasons discussed in the results [17,20,21]. Due to the one-on-one nature of the interviews in this study, participants may have not wanted to seem “like a bad parent” when answering questions on willingness to reduce risk, which may have influenced their responses. Additionally, we cannot discern what statements may be wishful thinking, or accurately predict the degree of follow-through that parents will have with risk-reduction steps until we begin returning risk results and monitoring parent behavior.

Systemic issues such as lack of access to affordable healthy food, safe places for physical activities, and health care were expected responses from participants as common barriers. These issues are not new, as they have been discussed previously in the literature as prevalent limitations for minority and underrepresented communities [20,21]. Perhaps the most notable barrier that participants reported was related to existing behavioral health issues in children. Many of the participants that had children with behavior difficulties reported this was their greatest barrier to implementing risk-reduction steps. Given that behavioral or psychiatric problems are common in childhood, with high prevalence of ADHD (9.4%) [22], behavioral conduct (7.4%), and anxiety problems (7.1%) [23], behavioral health clearly needs to be taken into consideration when trying to come up with strategies for risk reduction in these children. Utilizing the information here, health care providers planning on returning these types of risk reports to children should proactively find ways to provide resources and support to families to overcome these barriers, ensuring the child has a fair chance of reducing their risk.

Participants interviewed in this study generally agreed the reports were highly valuable and would be helpful not only for informing their child’s care, but also to the rest of their immediate family. Participants felt being able to review the risk report with their child’s PCP would make them feel the most comfortable and confident in their understanding. When receiving these results and report, participants emphasized what mattered most was having a resource they could trust to review the report with and ask questions. Additionally, participants were concerned about making sure their child’s PCP has knowledge of the report to watch for signs of disease throughout their child’s development. Knowing this is essential for planning for the return of polygenic results in the greater eMERGE 4 study. It will be critical that providers build trust with parents so they may address the questions and concerns appropriately. Looking at past research, there may be some burdens and barriers to PCPs to provide this extra care [24,25]. Our study shows this is something the patient requires to fully understand the implications of a polygenic risk report.

There are limitations to this study. One limitation is our sample population, as our findings were limited to forty parent/guardian interviews. We sampled participants from two East Coast children’s hospitals in metropolitan areas which could reflect a sampling bias in the opinions and thoughts of our participant group. Participants were parents/legal guardians of children already enrolled in biobanks at CHOP or BCH and did not necessarily constitute a representative sample. In addition, a relatively high proportion (37.5%) had prior experience with genetic testing (see Table 1). Although interviews provide an opportunity to gain better insight into individual participant’s perspectives, the one-on-one nature of the interviews could have influenced participants to answer a certain way to avoid feelings of judgment. During the interviews, if participants did not offer an emotional response to the report, they were given a set of probes as examples, which potentially distorted perceptions and/or responses. Although relevant to only a small minority, this should be considered a potentially biasing limitation. Additionally, enrollment was restricted to those who could participate in a video conference call. A unique aspect of this study was recruiting individuals of typically underrepresented backgrounds. Moving forward, it will be imperative to continue to include perspectives from these groups to better understand the perspectives and levels of understanding regarding genetic risk result return.

In conclusion, while participants saw great value in receiving risk score reports such as this, the reports did not come without some confusion. Absolute risk scores were preferred, and factors such as health of the child, family history, and the low statistical value contributed to participants not fully understanding the high risk associated with the report. When trying to create best practices to return these results to the pediatric population, we will need to utilize PCPs to aid in the return of results process, create educational materials to aid in understanding, and strategize ways to overcome systemic barriers found in the lives and neighborhoods of these individuals. Overall, this study provides an improved insight into implementing the return of genomic risk scores to pediatric patients in a clear, understandable, and fair way.

## Figures and Tables

**Table 1:** Interviewee Demographics.

	*N*	Percent		*N*	Percent
Total Interviewed	40	100.0%		40	100.0%
**Interview Site**			**Condition on Report**		
CHOP	20	50.0%	Type 2 Diabetes	20	50.0%
BCH	20	50.0%	Asthma	20	50.0%
**Type of Risk on Report**			**Gender**		
Relative	20	50.0%	Female	39	97.5%
Absolute	20	50.0%	Male	1	2.5%
**Race**			**Education**		
Black/African American	26	65.0%	Some High School	1	2.5%
White	3	7.5%	High School	7	17.5%
More than one race	11	27.5%	Post High School Training other than college	1	2.5%
**Ethnicity**			Some College	16	40.0%
Hispanic	9	22.5%%	Bachelor’s Degree	6	15.0%
Not Hispanic	31	77.5%%	Master’s Degree	8	20.0%
**Insurance**			Doctoral Degree	1	2.5%
Current of former employer or union	21	52.5%	**Age**		
CHIP	4	10.0%	21–30	3	7.5%
Medicaid, medical assistance, or any kind of government assistance	13	32.5%	31–40	20	50.0%
Other Source	2	5.0%	41–50	15	37.5%
**Genetic Experience**			51–60	2	5.0%
Previous Experience	15	37.5%			
No Experience	24	60.0%			
Did not Answer	1	2.5%			

**Table 2:** Domains and Themes.

Domains	Themes
**Understanding of Report**	Errors in perceiving risk scores
	Absolute risk framing was preferred
	Absolute risk tended to be perceived as low
	Several factors contributed to risk perception such as family history, trust in PCP, and medical complexity of the child
**Risk Reduction Steps**	Parents are willing to take risk-reduction steps
	Behavioral factors, diet/lifestyle, and lack of resources are barriers to taking risk reduction steps
**Reactions to Report**	Negative emotions were felt in response to the report including anxiety, worry and confusion
	Positive emotions were felt in response to the report including curiosity, relief, and confidence
	Most participants felt negative emotions at first, but by the end of the session felt positive ones
**Value of the Report**	Report prepared participants to take action to reduce risk
	High value of primary care providers explaining the results with participants

**Table 3:** Risk Perception.

		*N*	Percent
**Total Interviewed**		40	100.00%
**Overall**			
**Report Liked Best**	Absolute Risk	20	50.00%
	Relative Risk	15	37.50%
	Neither	5	12.50%
**Most Helpful Report**	Absolute Risk	19	47.50%
	Relative Risk	14	35.00%
	Neither	7	17.50%
**Most Confusing Report**	Absolute Risk	6	15.00%
	Relative Risk	7	17.50%
	Neither	27	67.50%

## Data Availability

To protect patient privacy, the dataset generated and analyzed during the current study may be available from the corresponding author on reasonable request contingent on a data use agreement.

## Supplementary Materials

The following are available online at https://www.mdpi.com/article/10.3390/jpm12121945/s1, Figure S1: Asthma Interview Guide, Figure S2: Type 2 Diabetes Interview Guide, Table S1: Risk Preferences.

## References

[1] Maas P., Barrdahl M., Joshi A.D., Auer P.L., Gaudet M.M., Milne R.L., Schumacher F.R., Anderson W.F., Check D., Chattopadhyay S. (2016). Breast Cancer Risk From Modifiable and Nonmodifiable Risk Factors Among White Women in the United States. JAMA Oncol..

[2] Schumacher F.R., Al Olama A.A., Berndt S.I., Benlloch S., Ahmed M., Saunders E.J., Dadaev T., Leongamornlert D., Anokian E., Cieza-Borrella C. (2018). Association analyses of more than 140,000 men identify 63 new prostate cancer susceptibility loci. Nat. Genet..

[3] Sharp S.A., Rich S.S., Wood A.R., Jones S.E., Beaumont R.N., Harrison J.W., Schneider D.A., Locke J.M., Tyrrell J., Weedon M. (2019). Development and Standardization of an Improved Type 1 Diabetes Genetic Risk Score for Use in Newborn Screening and Incident Diagnosis. Diabetes Care.

[4] Sordillo J.E., Lutz S.M., Jorgenson E., Iribarren C., McGeachie M., Dahlin A., Tantiria K., Kelly R., Lasky-Su J., Sakornaakolpat P. (2021). A polygenic risk score for asthma in a large racially diverse population. Clin. Exp. Allergy.

[5] Silarova B., Sharp S., Usher-Smith J.A., Lucas J., Payne R.A., Shefer G., Moore C., Girling C., Lawrence K., Tolkien Z. (2019). Effect of communicating phenotypic and genetic risk of coronary heart disease alongside web-based lifestyle advice: The INFORM Randomised Controlled Trial. Heart.

[6] Hollands G.J., French D.P., Griffin S.J., Prevost A.T., Sutton S., King S., Marteau T. (2016). The impact of communicating genetic risks of disease on risk-reducing health behaviour: Systematic review with meta-analysis. Bmj.

[7] Kahneman D., Slovic P., Tversky A. (1982). Judgement under Uncertainty: Heuristics and Biases.

[8] Tversky A., Kahneman D. (1974). Judgment under uncertainty:heuristics and biases. Science.

[9] Tversky A., Kahneman D. (1981). The framing of decisions and the psychology of choice. Science.

[10] Nightingale S.D. (1987). Risk preference and laboratory test selection. J. Gen. Intern. Med..

[11] Maynard C., Fisher L.D., Passamani E.R., Pullum T. (1986). Blacks in the coronary artery surgery study (CASS): Race and clinical decision making. Am. J. Public Health.

[12] Garcia-Retamero R., Galesic M. (2010). How to reduce the effect of framing on messages about health. J. Gen. Intern. Med..

[13] Huhn J.M., Potts C.A., Rosenbaum D.A. (2016). Cognitive framing in action. Cognition.

[14] Kwak Y., Huettel S. (2018). The order of information processing alters economic gain-loss framing effects. Acta Psychol. (Amst).

[15] Lewis A.C.F., Green R.C. (2021). Polygenic risk scores in the clinic: New perspectives needed on familiar ethical issues. Genome Med..

[16] Torkamani A., Wineinger N.E., Topol E.J. (2018). The personal and clinical utility of polygenic risk scores. Nat. Rev. Genet..

[17] Persky S., Yaremych H.E., Goldring M.R., Ferrer R.A., Rose M.K., Hollister B.M. (2021). Investigating the Efficacy of Genetic, Environmental, and Multifactorial Risk Information When Communicating Obesity Risk to Parents of Young Children. Ann. Behav. Med..

[18] Hillard M.E., Riekert K.A., Ockene J.K., Pbert L. (2018). The Handbook of Health Behavior Change.

[19] Collins R.E., Wright A.J., Marteau T.M. (2011). Impact of communicating personalized genetic risk information on perceived control over the risk: A systemic review. Genet. Med..

[20] Fisher E.B., Fitzgibbon M.L., Glasgow R.E., Haire-Joshu D., Hayman L.L., Kaplan R.M., Nanney M.S., Ockene J. (2011). Behavior matters. Am. J. Prev. Med..

[21] Patel N., Ferrer H.B., Tyrer F., Wray P., Farooqi A., Davies M.J., Khunti K. (2017). Barriers and Facilitators to Healthy Lifestyle Changes in Minority Ethnic Populations in the UK: A Narrative Review. J. Racial. Ethn. Health Disparities.

[22] Danielson M.L., Bitsko R.H., Ghandour R.M., Holbrook J.R., Kogan M.D., Blumberg S.J. (2018). Prevalence of Parent-Reported ADHD Diagnosis and Associated Treatment Among U.S. Children and Adolescents, 2016. J. Clin. Child Adolesc. Psychol..

[23] Ghandour R.M., Sherman L.J., Vladutiu C.J., Ali M.M., Lynch S.E., Bitsko R.H., Blumberg S. (2019). Prevalence and Treatment of Depression, Anxiety, and Conduct Problems in US Children. J. Pediatr..

[24] Mikat-Stevens N.A., Larson I.A., Tarini B.A. (2015). Primary-care providers’ perceived barriers to integration of genetics services: A systematic review of the literature. Genet. Med..

[25] Pet D.B., Holm I.A., Williams J.L., Myers M.F., Novak L.L., Brothers K.B., Wiesner G., Clayton E. (2019). Physicians’ perspectives on receiving unsolicited genomic results. Genet. Med..

