# Supplementary material for: Attitudes among Parents towards Return of Disease-Related Polygenic Risk Scores (PRS) for Their Children
Source: J Pers Med. 2022 Nov 23;12(12):1945. doi: 10.3390/jpm12121945 (PMC9786589; doi:10.3390/jpm12121945)
Supplement: Supplementary file 1 [file jpm-12-01945-s001.zip › Supplemental Table.pdf]

Supplemental Table: Risk Perception Comparison

		N	Percent			*N*	Percent
**Total Interviewed**		20	100.00%			20	100.00%
**BCH**				**CHOP**			
**Report Liked Best**	Absolute Risk	15	75.00%	**Report Liked Best**	Absolute Risk	5	25.00%
	Relative Risk	4	20.00%		Relative Risk	11	55.00%
	Neither	1	5.00%		Neither	4	20.00%
**Most Helpful Report**	Absolute Risk	14	70.00%	**Most Helpful Report**	Absolute Risk	5	25.00%
	Relative Risk	4	20.00%		Relative Risk	10	50.00%
	Neither	2	10.00%		Neither	5	25.00%
**Most Confusing Report**	Absolute Risk	2	10.00%	**Most Confusing Report**	Absolute Risk	4	20.00%
	Relative Risk	5	25.00%		Relative Risk	2	10.00%
	Neither	13	65.00%		Neither	14	70.00%
**Type 2 Diabetes**				**Asthma**			
**Report Liked Best**	Absolute Risk	7	35.00%	**Report Liked Best**	Absolute Risk	14	70.00%
	Relative Risk	10	50.00%		Relative Risk	4	20.00%
	Neither	3	15.00%		Neither	2	10.00%
**Most Helpful Report**	Absolute Risk	8	40.00%	**Most Helpful Report**	Absolute Risk	11	55.00%
	Relative Risk	7	35.00%		Relative Risk	7	35.00%
	Neither	5	25.00%		Neither	2	10.00%
**Most Confusing Report**	Absolute Risk	4	20.00%	**Most Confusing Report**	Absolute Risk	2	10.00%
	Relative Risk	2	10.00%		Relative Risk	5	25.00%
	Neither	14	70.00%		Neither	13	65.00%
