# Supplementary material for: Attitudes among Parents towards Return of Disease-Related Polygenic Risk Scores (PRS) for Their Children
Source: J Pers Med. 2022 Nov 23;12(12):1945. doi: 10.3390/jpm12121945 (PMC9786589; doi:10.3390/jpm12121945)
Supplement: Supplementary file 1 [file jpm-12-01945-s001.zip › Supplementary File 1 Asthma Interview Guide.pdf]

IRB 20-018128

Asthma Interview Guide:

Parents' perceived value of genomic risk estimates in children

Introduction & Description of the Study

[Direction: Read the following to the participant.]

Thank you so much for your interest in this eMERGE Study - Parents' perceived value of genomic risk estimates in children. I really appreciate your time and I am interested in your point of view. Please let me know at any time if you have questions. Any information you provide during this interview is voluntary – meaning that you don't have to do anything you don't want to do.

- Before we start officially, do you have any questions?
  - *[If Yes, record question(s) and answer in notes.]*
- Are you ready to begin with the interview?
  - *[If Yes, continue.*
  - *If No, ask the participant if they need a few minutes or if they are not interested in continuing with the interview today. Document response.]*

We contacted you for this study because your child [CHILD'S NAME] is enrolled in the Center for Applied Genomics (CAG) at the Children's Hospital of Philadelphia (CHOP) Biobank. At the time [CHILD NAME] was enrolled in the biobank, permission was given for you to be contacted in the future to learn about other studies.

In this study, we hope to learn about how best to give parents results about their <u>child's risk</u> for getting a disease based on their <u>child's genetics</u>. We call this a "genetic risk result". We want to learn what parents think about getting a genetic risk result on their child so we can find the best ways to return these results to parents in the future for those who are interested. We will ask you questions about how you would feel about getting genetic risk results and how those results could impact your child's medical care.

Your input in this interview will help us figure out the best way to give these results to parents in the future. Your child has NOT and will NOT be tested as part of this study and you will NOT recieve an actual (real) genetic risk report for your child.

Instructions

[Read the following to the participant.]

I would like to go over some of the specifics of the interview with you at this point.

You may recall that we sent you a “hypothetical report” (a report that is not real). If you don’t remember what you read, or you didn’t have a chance to read it; that is ok. You will have time to look at the report during the interview.

For this interview, I want you to pretend that you got this report on your child [CHILD’S NAME]. Remember this is not a real report on [CHILD’S NAME] – it is a fake report.

I will ask you questions about what you think about the whole report and then what you think about certain parts of the report. It is ok if you don’t know a lot about genetics. We want to learn what people who are not genetic experts think about genetic risk results. So, what we learn from you will be very helpful to researchers.

If you don't understand a question, why a question is asked, a word, or any part of the interview, please let me know. Also, if there is part of the interview that you do understand, but that you think other people may not understand, please let me know. Again, what we learn from you will be very helpful for the research team.

Everything you tell me will be kept private. This means what you tell us will only be shared with our research team. When we write our report on this interview, nothing in the report will identify you.

Please be honest with your responses. You can say what you want, nothing will hurt my feelings, and nothing you say will affect your or your child’s medical care. We will record this conversation in order to study your responses to write our report. I will tell you when I start the recording. Please remember, you don’t have to talk about anything you do not want to talk about. You don’t have to answer any question that you don’t want to, and you can stop this interview at any time.

Confirmation of informed consent

- Do you have any questions about taking part in this study?
  - *[If Yes, record question(s) and answer in notes.]*
- Are you still willing to do this interview?
  - (If No) Thank you for your time and interest. Feel free to reach out to us if you change your mind and want to do this interview in the future.
  - (If Yes) Thank you. Is it okay if I start recording now?

*[Ask the participant to silence their phone (ok if they don’t want to) and turn off their screen. *If the participant is using their phone for the interview, the phone and screen will need to stay on for the entire study. Ask if they are in a private, quiet area (or if they can move to one). If they are not in a private area, record in notes. Start recording. Verify recording started.]*

[State after recording starts and before interview starts:

1. Participant’s study ID number

2. Interviewer’s name

3. Date of interview

4. Type of report*

5. Time of interview

6. Type of device the participant will use to view reports during the interview.]

*Type of report first seen/sent to participant: 1. Asthma Absolute Risk 2. Asthma Relative Risk 3. Type 2 Diabetes Absolute Risk 4. Type 2 Diabetes Relative Risk

(what we are focusing on here is ASTHMA).

Review of materials sent to participant

1. Did you have time to read the hypothetical report that was sent to you? This is the pretend report that is not real.
    a. (If Yes) Thank you, I will share my screen with the report in case you want to look at it during the interview.
    b. (If No) I will share my screen with the report and give you a few minutes to read it. Please let me know when you are done reading it.

[Show Slide #3. Give the participant 3 minutes and check if the participant needs more time. Document amount of time participant spent reviewing the report.]

2. Do you have any questions about the report before we start the interview? *[If Yes, record question(s) and answer in notes.]*

Assess Understanding of Risk

[Continue to share your screen with the 1st report visible. Verbally confirm that this is report sent home to participant. Verbally note if relative or absolute risk report.}

You received one hypothetical report prior to the interview. We would like to find out what you think of the report and what it means to you.

3. What do you think the test results on the hypothetical report mean for your child's health?

4. Based on the hypothetical report, what do you feel your child's risk would be for getting Asthma in their lifetime? *[Show Likert scale on screen, Slide #4.]*
   ☐very low, ☐low, ☐average, ☐high, ☐very high

    a. Can you explain why you chose ________? *[Explore how they came to that conclusion.]*

5. Do you think genetic risk testing could help you understand the risk in your family for getting a disease?
    a. (If No) Why?
    b. (If Yes) How?

6. Did you find anything on the report confusing?
    a. (If Yes) What was confusing?
    b. Why was it confusing?

7. Did you find anything on the report helpful?

a. (If Yes), What was helpful?

b. Why was it helpful?

[Show second report that participant has not seen yet. Verbally document which report now is shown on screen. Slide #5]

Here is a second hypothetical or made-up report about the same test results that is a little bit different. Please take a few minutes to read the second report and let me know when you have read it.

[Record amount of time the participant takes to review the report.]

8. What do you think the test results on the hypothetical report mean?

9. Based on the hypothetical report, what do you feel your child's risk would be for getting Asthma in their lifetime? *[Show Likert scale on screen. Slide #6]*
(☐very low, ☐low, ☐average, ☐high, ☐very high)

    a. Can you explain why you chose _________? *[Explore how they came to that conclusion*

10. Did you find anything on the second report confusing?

    a. (If Yes) What was confusing?

    b. Why was it confusing?

    c. What could be changed to make it less confusing?

11. Did you find anything on the second report helpful?

    a. (If Yes) What was helpful?

    b. Why was it helpful?

Assessing and Comparing the Reports

Now I'd like you to think about how the 2 reports compare to each other. *[Sildes #7 and #8]*

12. Was one report more helpful than another?

    a. (If Yes) Which one was more helpful?

    b. Why was it more helpful?

13. Was one report more confusing than another?

    a. (If Yes) Which one was more confusing?

    b. Why was it more confusing?

14. Which report did you like the best?

    a. Why did you like it more?

15. Is there anything else that could be in the report that would help you understand the genetic risk results and what they mean?
    a. (If Yes) Please explain.
16. Is there anything that could be taken out of the reports that was confusing or is not needed?
    a. (If Yes) Please explain.
17. Is there anything about the report that makes it hard to read?
    a. (If Yes) Please explain.
18. Before we move on to the next set of questions, is there anything else you would like to tell me about the report(s)?
    a. (If Yes) Please explain.

Assess Value of the Genetic Risk and Risk Reduction Actions

[*Show first report again. Slide#9*]

19. Do you think there is value in you knowing genetic risk information about your child?
    a. (If Yes) What is the value?
    b. (If No) Why not?

20. How would you rate the value in your child's primary care doctor knowing genetic risk information about your child? *[Show Likert scale on screen. Slide #10 Two-part answer. Collect scale value & why.]*.
    (☐very low, ☐low, ☐average, ☐high, ☐very high)
    a. Can you explain why you chose ________? *[Explore how they came to that conclusion.]*
21. If your child's primary care doctor has access to this report in the medical record, how would you rate the value in being able to review this report with them? *[Show Likert scale on screen. Slide#11 Two-part answer. Collect scale value & why.]*.
    (☐very low, ☐low, ☐average, ☐high, ☐very high)
    a. Can you explain why you chose ________? *[Explore how they came to that conclusion.]*
22. ~~Should we worry that people might have reasons not to trust the risk results on the reports?~~
    a. ~~(If yes) What do you think those reasons could be? *[Prompt: Lack of trust in the medical / scientific community, lack of trust in genetics, lack of trust in clinical research]*~~

Assess Utility of the Genetic Risk and Risk Reduction Actions

[Slide #12] Pretend that your child's primary care doctor has seen this report and tells you that there are things that can be done that could help to reduce your child's risk for getting Asthma (and/or making their Asthma worse).

23. How likely are you to try to get your child to do each of these changes a doctor suggests? *Read the list below and document the answer. Show the Likert scale. Slide #13]*

    a. Change or limit exposure to infections. (☐not likely, ☐somewhat likely, ☐likely, ☐most likely, ☐highly likely)

    b. Change or limit exposure to allergens such as mold, pet dander, dust, and cockroaches, and irritants such as smoke and cold air. (☐not likely, ☐somewhat likely, ☐likely, ☐most likely, ☐highly likely)

    c. Use preventative therapy or asthma drug as prescribed (☐not likely, ☐somewhat likely, ☐likely, ☐most likely, ☐highly likely)

    d. Get a yearly flu vaccine. (☐not likely, ☐somewhat likely, ☐likely, ☐most likely, ☐highly likely)

24. There may be reasons why making changes to lower the risk that your child would get the condition listed in the report hard. We call these "barriers." Can you think of barriers that would make it hard to make the changes suggested in a report like this? *[Slide #14]*

    a. *[(If Yes) Expand on why/how and document responses.]*

    b. *[(If No) Go to next question.]*

[Once they have brainstormed on barriers or said 'No', read the barriers below that they didn't mention and document choice(s).]

25. In addition to the barriers you mentioned, would any of the following also be barriers to try to make changes suggested in a report like this? *[Show Asthma Barriers on screen, Slides#15 and #16. For any 'Yes' response, follow-up with 'How/why do you think xx is a barrier?' Document responses.]*

    a. ☐ Home exposures such as dust, tobacco smoke, pet dander, mold, pests, such as cockroaches,

    b. ☐ Community (ex: neighborhood, school) exposures to dust, smoke, etc.,

    c. ☐ Difficulty traveling to/from appointments,

    d. ☐ Lack of support from family, friends, or your community

    e. ☐ Lack of education materials/resources,

f. ☐ Not having a detailed plan with the doctor,

g. ☐ No insurance or problems with your type of insurance,

h. ☐ New costs associated with doing what your doctor suggests,

   i. Can you think of other examples of costs that could be barriers? *[(If Yes,) document responses.]*

i. ☐ Lack of time,

j. ☐ Behavioral health issues that impact your child, such as autism or ADHD,

k. ☐ It would be hard for your child to feel 'different' from their peers,

l. ☐ Lack of motivation or cooperation from your child

m. ☐ Other?

26. Can you think of some things that would help you follow the changes suggested by your child's doctor?

   a. (If Yes) Please tell me your idea(s) in as much detail as you can. *[Expand on why/how and document responses.]*

27. In addition to the ideas you just mentioned, would any of the following be things that could help you follow the changes suggested by your child's doctor? *[Show Resources on screen. Slides#17 and #18.]*

   a. ☐ Removal or reduction of home exposures such as dust, mold, pets and pests

   b. ☐ A family member in your house would stop smoking

   c. ☐ Guaranteed or affordable travel to/from appointments

   d. ☐ Support from family, friends, or community (ex. Church, child's school, peer group)

   e. ☐ Availability of and access to education materials/resources,

   f. ☐ A detailed plan with doctor,

      i. (If Yes) What does detailed mean to you?

   g. ☐ Insurance coverage and/or free services,

   h. ☐ Behavioral health services or support at home, such as behavior therapy

   i. ☐ Other?

<u>Assess Emotional Response to the Genetic Risk Reports</u>

28. How did this hypothetical report on your child make you feel? *[Show visual, Slide #19]*

    a. *[Probe (if needed)]*: For example, did you feel anxious, frustrated, curious, surprised, worried, overwhelmed, confused, confident, neutral?

    b. *[(If Yes) Expand on why/how and document responses.]*

29. If you got this report about your child and needed to tell somebody about what you learned from the report, how would you explain what you learned?

30. Should we worry that people might have reasons to not trust the risk results on the reports?

    a. (If yes) What do you think those reasons could be? *[Prompt: Lack of trust in the medical / scientific community, lack of trust in genetics, lack of trust in clinical research]*

31. Does seeing this made-up report affect your current interest in genetic testing? *[Show Likert scale, Slide #20]*

    (☐not at all, ☐a little bit, ☐somewhat, ☐quite a bit, ☐extremely)

    a. Can you explain why you chose _________? *[Explore how they came to that conclusion.]*
    b. Did your interest go up or down? *[Record Y/N]*

32. Is there anything else about this set of questions about genetic risk/testing you would like to talk to us about now?

Wrap Up *[Slide#21]*

33. Do you have anything to add that we have not discussed? Or something you would like to discuss in more detail?
34. Is there anything to take out or put in the report?
    a. (If Yes) Why do you think we should make those changes? *[Prompt: readability, formatting, confusing, etc.]*
35. Is there anything to take out or put in the interview?
    a. (If Yes) Why do you think we should make those changes? *[Prompt: readability, formatting, confusing, etc.]*
36. Do you have any other questions or comments?

I just want to remind you, these were mock reports. Your child has NOT and will NOT be tested as part of this study and you will NOT receive a genetic risk report for your child.

I have finished the interview questions about the reports and I will now stop the recording.

Demographic Information

We would like to ask some questions to learn more about you.

37. What is your age?

38. What is your gender?

a. ☐ Male

b. ☐ Female

c. ☐ Other

d. ☐ Prefer not to answer.

39. Do you identify as Latino/a or Hispanic?
    a. ☐ Yes

    b. ☐ No

    c. ☐ Prefer not to answer.

40. Which one or more of the following races do you identify? Select all that apply.

    a. ☐ American Indian or Alaska Native

    b. ☐ Asian

    c. ☐ Black or African American

    d. ☐ Middle Eastern or North African

    e. ☐ Native Hawaiian or Pacific Islander

    f. ☐ White

    g. ☐ Other

    h. ☐ Prefer not to answer.

41. What is the highest grade or year of school you completed? (One response.)

    a. ☐ Never attended school or only kindergarten.

    b. ☐ Grade school (grades 1 to 8)

    c. ☐ Some high school (grades 9 to 12)

    d. ☐ High school graduate or GED

    e. ☐ 1 to 3 years after high school (Some college, Associate's degree, or technical school)

    f. ☐ College 4 years or more (College graduate)

    g. ☐ Advanced degree (Master's, Doctorate, etc.)

    h. ☐ Prefer not to answer

42. Is your child currently covered by any of the following types of health insurance or health coverage plans? Y/N for each

a. Insurance through a current or former employer or union (through you or another family member)

b. Insurance purchased directly from an insurance company (by you or another family member)

c. The Children's Health Insurance Program (CHIP)

d. Medicaid, Medical Assistance, or any kind of government-assistance plan for those with low incomes or a disability

e. TRICARE or other military health care

f. Indian Health Service

g. Any other type of health insurance or health coverage plan - specify

43. Do you have any experience with genetic testing?

    a. (If Yes) Can you explain your experience?

44. Could you confirm your current mailing address?

45. Could you confirm your current e-mail address?

Again, thank you so much for talking with me today! Your thoughts and answers will help us to make genetic risk reports that people will find easier to read and understand. Feel free to call us if you have any further questions (give phone number). We will mail / email you the gift debit card for completing the study. *[Complete confirmation / receipt...TBD]*
